# Prevalence of Symptomatic Heart Failure with Reduced and with Normal Ejection Fraction in an Elderly General Population–The CARLA Study

**DOI:** 10.1371/journal.pone.0059225

**Published:** 2013-03-15

**Authors:** Daniel Tiller, Martin Russ, Karin Halina Greiser, Sebastian Nuding, Henning Ebelt, Alexander Kluttig, Jan A. Kors, Joachim Thiery, Mathias Bruegel, Johannes Haerting, Karl Werdan

**Affiliations:** 1 Institute for Medical Epidemiology, Biostatistics, and Informatics Martin-Luther-University Halle-Wittenberg, Halle (Saale), Germany; 2 Department of Medicine III, University Clinics Halle (Saale), Martin-Luther-University Halle-Wittenberg, Halle (Saale), Germany; 3 German Cancer Research Centre Division of Cancer Epidemiology, Heidelberg, Germany; 4 Department of Medical Informatics, Erasmus University Medical Centre, Rotterdam, The Netherlands; 5 Institute of Laboratory Medicine, Clinical Chemistry and Molecular Diagnostics, University of Leipzig, Leipzig, Germany; University Hospital of Würzburg, Germany

## Abstract

**Background/Objectives:**

Chronic heart failure (CHF) is one of the most important public health concerns in the industrialized world having increasing incidence and prevalence. Although there are several studies describing the prevalence of heart failure with reduced ejection fraction (HFREF) and heart failure with normal ejection fraction (HFNEF) in selected populations, there are few data regarding the prevalence and the determinants of symptomatic heart failure in the general population.

**Methods:**

Cross-sectional data of a population-based German sample (1,779 subjects aged 45–83 years) were analyzed to determine the prevalence and determinants of chronic SHF and HFNEF defined according to the European Society of Cardiology using symptoms, echocardiography and serum NT-proBNP. Prevalence was age-standardized to the German population as of December 31st, 2005.

**Results:**

The overall age-standardized prevalence of symptomatic CHF was 7.7% (95%CI 6.0–9.8) for men and 9.0% (95%CI 7.0–11.5) for women. The prevalence of CHF strongly increased with age from 3.0% among 45–54- year-old subjects to 22.0% among 75–83- year-old subjects. Symptomatic HFREF could be shown in 48% (n = 78), symptomatic HFNEF in 52% (n = 85) of subjects with CHF. The age-standardized prevalence of HFREF was 3.8 % (95%CI 2.4–5.8) for women and 4.6 % (95%CI 3.6–6.3) for men. The age-standardized prevalence of HFNEF for women and men was 5.1 % (95%CI 3.8–7.0) and 3.0 % (95%CI 2.1–4.5), respectively. Persons with CHF were more likely to have hypertension (PR = 3.4; 95%CI 1.6–7.3) or to have had a previous myocardial infarction (PR = 2.5, 95%CI 1.8–3.5).

**Conclusion:**

The prevalence of symptomatic CHF appears high in this population compared with other studies. While more women were affected by HFNEF than men, more male subjects suffered from HFREF. The high prevalence of symptomatic CHF seems likely to be mainly due to the high prevalence of cardiovascular risk factors in this population.

## Introduction

Chronic heart failure (CHF) is one of the most important health concerns in the industrialized world. Whereas survival with CHF has improved over the last decades, its prevalence and incidence are steadily increasing [1].

According to the *European Society of Cardiology* (ESC), heart failure is defined as a syndrome that consists of symptoms or signs typical of heart failure, such as shortness of breath or raised jugular venous pressure, and objective evidence of a structural or functional abnormality of the heart at rest, e.g., in an echocardiogram. [2]. Additionally, the diagnosis of heart failure is supported by the laboratory analysis of natriuretic peptides such as NT-proBNP or BNP which reflect neurohormonal activation. The diagnostic value of these biomarkers is characterized by a high negative predictive value for the exclusion of LV dysfunction. Furthermore, the natriuretic peptide concentration in plasma/serum (particularly B-type natriuretic peptide, BNP) correlates with left ventricular (LV) function and mass [3], [4].

The objective evidence of normal systolic LV function accompanied by an impaired diastolic function in about one third of patients with clinical signs of heart failure led to the differentiation between heart failure with reduced ejection fraction (HFREF) and heart failure with normal ejection fraction (HFNEF) [5], [6].

Systolic dysfunction is primarily considered as reduced contraction ability of the heart muscle resulting in a decreased ejection fraction. In contrast, diastolic dysfunction is characterized by a disturbed relaxation and dilatation of the left ventricle after the contraction. The filling of the ventricles is slow or incomplete unless atrial blood pressure rises, but the ejection fraction is preserved. Although cardiac structure and function differ substantially in systolic and diastolic dysfunction, the clinical signs and symptoms are nearly the same [7]. However, clinical characteristics such as sex, hypertension, chronic obstructive pulmonary disease (COPD) or atrial fibrillation differ between patients with decompensated HFNEF and HFREF [8].

Despite a wide variation in the reported prevalence of chronic heart failure, published data suggest that the prevalence has been steadily increasing over the past decades [9]. Overall, the prevalence of heart failure is estimated to be 1–2% in the industrialized world and the incidence is estimated to be 5–10 per 1,000 persons per year [10]. Heart failure is hardly found in subjects younger than 50 years, but in subjects above 50 years the prevalence increases progressively with age. According to analyses from the Rotterdam Study, the prevalence of symptomatic heart failure increases from 0.5% among subjects aged 55–64 years to almost 14% among subjects aged 85–94 years [11]. A population-based study from the USA shows an increase of CHF prevalence from 0.7% in the lowest age group (45–54 years) to more than 8% in subjects aged over 74 years [12].

Many attempts have been made to describe the burden of CHF in the population. Although there are several studies estimating the prevalence of HFREF and HFNEF in selected populations [10]–[16] there are few data regarding the prevalence and the determinants of symptomatic HFREF as well as HFNEF in the general population. Moreover, there is a lack of data on the distribution of symptomatic CHF among the general population using natriuretic peptides as diagnostic markers. The main objective of this study was therefore to describe the burden of symptomatic CHF and the distribution of HFREF vs. HFNEF using a diagnostic approach including self-reported symptoms, echocardiographic information on systolic and diastolic function, and NT-proBNP level in an elderly general population with an especially high burden of adverse risk factors, such as hypertension, obesity and diabetes [17], [18]. Furthermore, we aimed to describe the association of hypertension, previous MI, coronary heart disease and obesity with CHF, HFREF and HFNEF.

## Methods

To determine the prevalence of overall CHF we used cross-sectional data from the baseline examination of the ***CA*** *rdiovascular* ***R*** *isk Factors,* ***L*** *iving and* ***A*** *geing in Halle* Study (CARLA Study), conducted between December 2002 and January 2006 in Halle (Saale) in Saxony-Anhalt. The CARLA Study is a German population-based prospective cohort study. Details of the study design and methods have been described elsewhere [19], [20]. The study population comprises 812 female and 967 male inhabitants of the city of Halle aged 45 to 83 years and the baseline response was 64%. Subjects were excluded if they were too ill to participate in the 4-hour examination. Furthermore, we did not include subjects living in nursing homes. An analysis of 373 non-respondents was carried out in order to obtain information on prevalent disease, selected behavioural factors and sociodemographic variables.

The study was approved by the Ethics Committee of the Medical Faculty of the Martin-Luther-University Halle-Wittenberg and by the State Data Privacy Commissioner of Saxony-Anhalt and conforms with the principles outlined in the Declaration of Helsinki [21]. All participants gave written informed consent. Data were collected with standardized, computer-assisted personal interviews and medical examinations by trained study nurses. To examine left ventricular (LV) function two-dimensional Doppler-echocardiography was performed in all subjects participating in the study by a physician who had been specifically trained and certified for this study. Serum levels of NT-proBNP were determined in all subjects from non-fasting venous blood samples after a 30 minute rest, using the Elecsys proBNP sandwich immunoassay (Roche Diagnostics).

### Definition of heart failure in the study population

**Figure 1:**
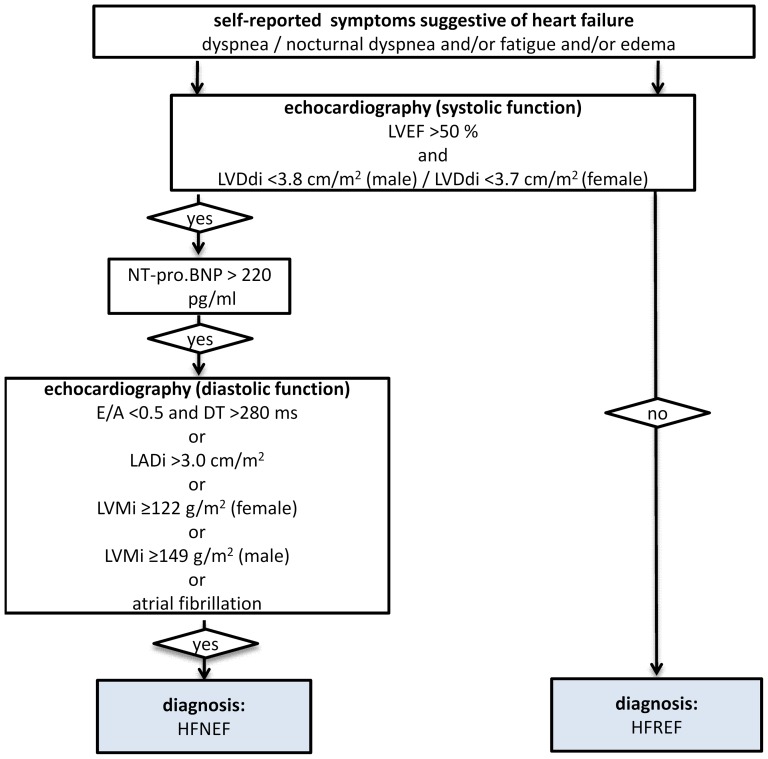
Algorithm to define symptomatic CHF.

The diagnosis of CHF was based on (a) self-reported symptoms suggestive of heart failure in combination with (b) an increase of the NT-proBNP serum concentration above 220 pg/ml and (c) on the evidence of systolic or diastolic dysfunction using echocardiographic parameters according to the recommendation of the ESC [22] (see Fig. 1). Information on symptoms of heart failure was obtained via standardized interview. Individuals were asked whether they suffer from dyspnoea, fatigue, or oedema. Furthermore, the severity of these symptoms was assessed in order to determine the NYHA classification.

The echocardiographic parameters LV ejection fraction (LVEF) and LV diameter index (LVDI) were used to determine the left ventricular systolic function. According to the recommendation of the ESC, systolic function was considered as normal or mildly reduced if LVEF was greater than 0.5 and LVDI was less than 3.8 cm/m^2^ in male subjects and less than 3.7 cm/m^2^ in female subjects [22], [23]. The LVEF was determined from the long parasternal axis and calculated according to the Teichholz equation. Diastolic function was determined based on the following parameters: ratio of peak early (E) to peak atrial (A) Doppler mitral valve flow velocity (E/A), deceleration time (DT), left atrial diameter index (LADI) and the sex-specific left ventricular mass index (LVMI). To calculate the LVMI we used the ASE-cube formula according to international guidelines [23], [24]. The cut-off values for these parameters are shown in Figure 1. For NT-proBNP we used a cut-off value of 220 pg/ml.

All subjects with self-reported symptoms and a reduced LV systolic function (LVEF ≤50% **or** LVDI ≥3.8 cm/m^2^ for males and ≥3.7 for females) were classified as having **heart failure with reduced ejection fraction** (HFREF). Those subjects with self-reported symptoms suggestive of heart failure, an elevated NT-proBNP (>220 pg/ml), and a reduced diastolic function (according to the above mentioned echocardiographic criteria) but a normal or only mildly reduced LV systolic function (LVEF >50% **and** LVDi <3.8 cm/m^2^ for males and <3.7 for females) were classified as having **heart failure with normal ejection fraction** (HFNEF) (see Figure 1).

### Definition of covariates

Hypertension was defined as mean systolic blood pressure (SBP) ≥140 mmHg, and/or mean diastolic BP ≥90 mmHg, and/or use of antihypertensive medication according to the anatomic therapeutic chemical classification system (ATC codes C02, C03, C07, C08, C09). Blood pressure measurement was performed in sitting position after a 5-minute resting phase using the OMRON HEM-705CP device. The average of the second and third of three measurements was used for further analyses. Prevalent myocardial infarction (MI) was defined as self-reported physician-diagnosed MI and/or definite MI by Minnesota code of the 10-second ECG [25]. ECGs were processed by the Modular ECG Analysis System (MEANS) [26] to obtain Minnesota codes. If the subject did not report a MI, but the Minnesota code indicated a definite MI, the ECG-based diagnosis was verified by a cardiologist. Coronary heart disease (CHD) was defined as definite MI, and/or self-reported coronary artery bypass graft, and/or self-reported percutaneous coronary intervention. Cardiovascular disease was defined as CHD, and/or self-reported physician-diagnosed stroke, and/or carotid surgery. Diabetes mellitus (DM) was defined as self-reported physician-diagnosed diabetes and/or use of antidiabetic medication (ATC code A10).

### Statistical analysis

All statistical analyses were performed using the Statistical Analysis Software (SAS 9.2, SAS Institute, Cary, NC, USA). Prevalences were directly age-standardized to the German standard population as of December 31st, 2005. The corresponding confidence intervals were calculated according to the recommendations of Fay and Feuer [27]. Overall means of risk factors were calculated using age-specific weights of the standard population. To describe the association between CHF and different determinants we used the prevalence ratio (PR) as effect measure. Therefore, we calculated generalized linear models with the SAS*®* procedure *PROC GENMOD* using the binomial distribution and the log link [28].

## Results

### Study population

The study population of the CARLA Study comprises 812 women and 967 men aged 45–83 years at baseline with a mean age (SD) of 63.8 (9.9) and 64.9 (10.2) years, respectively. The baseline characteristics of this population are shown in Table 1. Almost 75% of the study population showed hypertension. The prevalence of cardiovascular diseases was considerably higher in men than in women, while diabetes showed a similarly high prevalence in both sexes. Although the mean body mass index (BMI) was quite similar in both sexes, women were more likely to be obese than men (34% and 29%, respectively, with a BMI of 30 kg/m^2^ or more).

**Table 1:** Baseline characteristics of the CARLA Study population (2002–2006).

	Women (N = 812)			Men (N = 967)		
	N	Proportion^1^/Mean/Median	(95% Confidence Interval/IQR)	N	Proportion^1^/Mean/Median	(95% Confidence Interval/IQR)
Age (yrs)	812	63.8	(63.1–64.4)	967	64.9	(64.2–65.5)
SBP^2^ (mmHg)	812	140.4	(138.9–142.0)	966	145.4	(144.2–146.7)
DBP^2^ (mmHg)	812	83.5	(82.8–84.3)	966	86.9	(86.2–87.5)
Glucose (mmol/l)	807	5.78	(5.67–5.89)	960	6.13	(6.01–6.25)
Body mass index (kg/m^2^)	812	28.5	(28.1–28.9)	967	28.2	(27.9–28.4)
Waist-to-hip ratio (WHR)	812	0.88	(0.88–0.89)	967	1.0	(1.0–1.01)
Smoking^2^:						
Current	119	17.1	(14.0–20.8)	225	27.6	(23.8–31.9)
Past	140	18.1	(15.1–21.7)	496	46.9	(42.6–51.6)
Never	553	64.8	(59.3–70.8)	245	25.5	(22.2–29.3)
LV ejection fraction	777	0.65	(0.65–0.66)	902	0.62	(0.61–0.63)
LV mass (g/m^2^)	784	106.7	(104.7–108.6)	910	124.3	(119.5–129.2)
NT-proBNP (pg/ml)^3^	790	92.5	(51.7–158.3)	932	62.5	(33.5–140.7)
GFR_MDRD_ 4	800	88.6	(87.2–90.1)	967	96.0	(94.5–97.4)
**Disease prevalence:**						
Myocardial infarction	17	2.00	(1.00–3.00)	88	7.55	(5.91–9.20)
Stroke	27	2.99	(1.85–4.14)	42	3.57	(2.46–4.68)
Cardiovascular Disease (CVD)^5^	48	5.52	(3.92–7.13)	153	12.84	(10.7–14.9)
Hypertension^6^	608	72.0	(66.0–78.0)	789	78.8	(72.9–84.6)
Diabetes mellitus^7^	120	13.3	(10.9–15.7)	154	14.2	(11.9–16.6)

1 Proportions age-standardized to the age-distribution of the German standard population as of 31.12.2005, means weighted with age-specific weights of standard population (except for mean age)

2 SBP = systolic blood pressure, DBP = diastolic blood pressure, smoking = smoking tobacco products defined as cigarettes/cigars/pipes,

3 given as median and inter-quartile-range

4 glomerular filtration rate according to the simplified MDRD (Modification of Diet in Renal Disease) equation

5 CVD: including prevalent myocardial infarction, coronary artery bypass graft, percutaneous transluminal coronary angioplasty, stroke, carotid surgery

6 Hypertension defined as SBP >  = 140and/or DBP> = 90 mmHg, and/or use of antihypertensive medication by ATC code

7 Diabetes defined as self-reported physician-diagnosed diabetes mellitus and/or use of anti-diabetic medication by ATC code

### Prevalence of chronic heart failure

**Figure 2:**
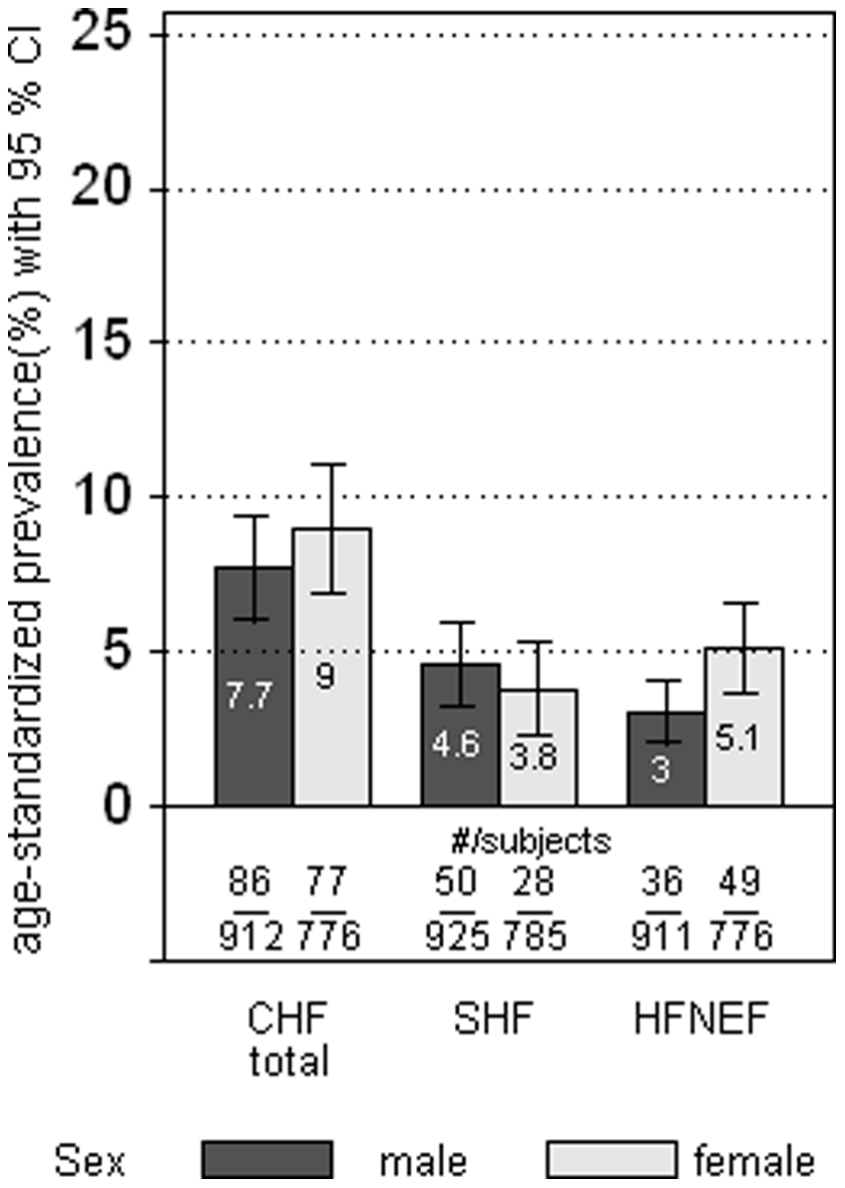
Age-standardized prevalence of symptomatic CHF, HFREF and HFNEF (different denominators are due to by differences in the number of subjects with missing data for HFREF and HFNEF).

Symptomatic CHF was diagnosed in 163 subjects (86 men and 77 women) resulting in an age-standardized prevalence of 8.3% (95%CI 7.0–9.8) in the total study group and 7.7% (95%CI 6.1–9.8) in men and 9.0% (95%CI 7.0–11.5) in women, respectively. The overall age-standardized prevalence of symptomatic HFREF and symptomatic HFNEF was 4.2% (95%CI 3.3–5.5) and 4.0% (95%CI 3.2–5.1), respectively. Overall, the prevalence of CHF did not differ between men and women. However, whereas more men were affected by symptomatic HFREF (age-adjusted prevalence ratio (PR) for male vs. female subjects 1.5 (95%CI 0.9–2.4)), more women were affected by symptomatic HFNEF, (age-adjusted PR for female vs. male subjects 1.8 (95%CI 1.2–2.7) (see Figure 2). Of all subjects with left ventricular dysfunction (either systolic or diastolic) 31% did not report symptoms suggestive of heart failure. Asymptomatic CHF was more common in men (36%) than in women (23%).

**Figure 3:**
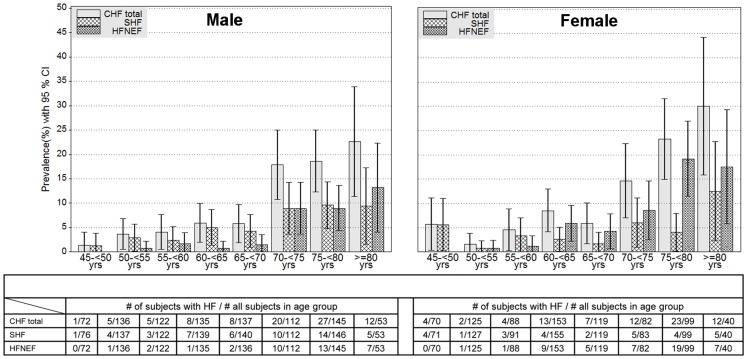
Age-specific prevalence of symptomatic CHF, HFREF and HFNEF by 5-year-age-groups.

The prevalence of symptomatic CHF strongly increased with age from 3.0% among 45–54- year-old subjects to 22.0% among 75–83- year-old subjects. Figure 3 shows the age-specific prevalence of overall symptomatic CHF, SHF and HFNEF for 5-year age groups in men and women. The prevalence of heart failure increased from 1.4% for men aged 45–54 years to 22.6% for men aged ≥75 years. For women, the prevalence rose from 5.7% in the lowest to 30.0% in the highest age group.

While men showed a lower LVEF than women (0.62% (SD 0.09) vs. 0.65% (SD 0.08)) women showed a higher NT-proBNP serum concentration (median 65.5 pg/ml (IQR 33.5–140.7) vs. 92.5 pg/ml (IQR 51.7–158.3)). Subjects with HFNEF showed the highest LVMI (mean 184.0 g/m2 (SD 51.9)). Subjects with HFNEF showed the highest NT-proBNP serum concentration (median 424.5 pg/ml (IQR 279.5–808.4)) followed by subjects with HFREF (259.3 pg/ml (IQR 86.2–846.1)). As Table 2 shows, about half of all subjects with SHF and HFNEF were in NYHA class II and about one third were in NYHA class III. More than 10% of all subjects with SHF could be classified as NYHA class IV and almost 18% of all subjects with HFNEF as NYHA class IV.

**Table 2:** NYHA-Classification among subjects with symptomatic SHF and HFNEF.

	HFREF			HFNEF		
	N	Proportion (95%CI)		N	Proportion (95%CI)	
NYHA II	42	53.8	(38.8–68.9)	43	50.6	(40.0–61.2)
NYHA III	28	35.9	(24.7–47.1)	27	31.8	819.5–44.1)
NYHA IV	8	10.3	(0.2–20.3)	15	17.6	(2.1–33.2)

**Figure 4:**
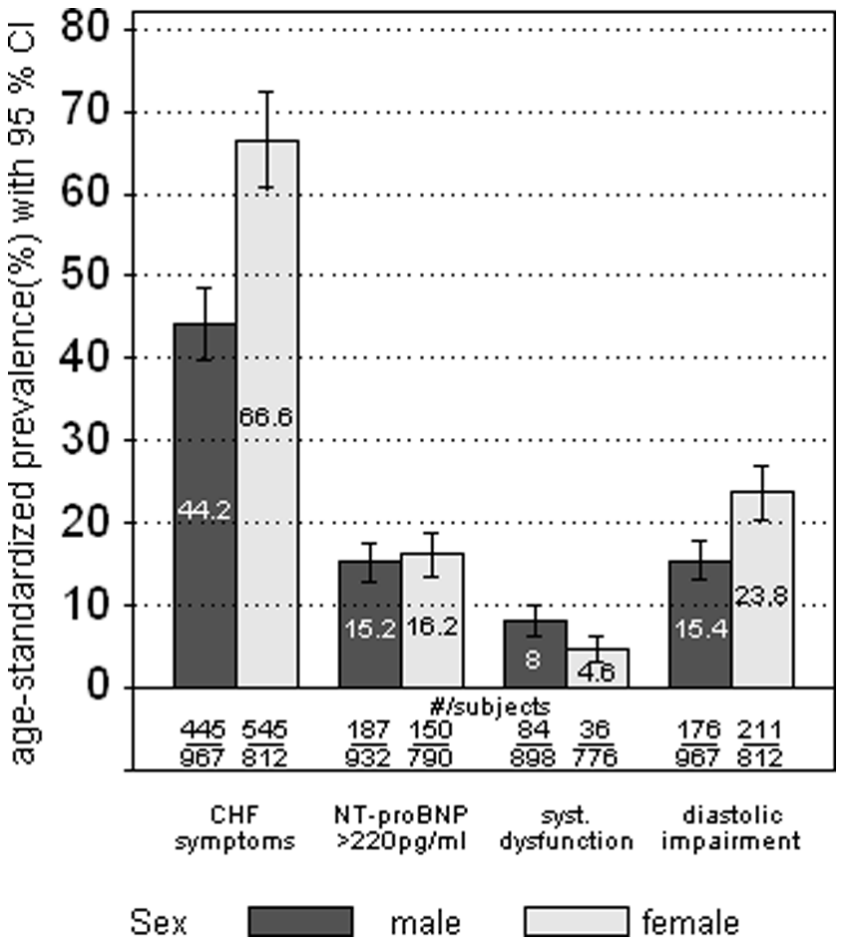
Age-standardized prevalence of self-reported symptoms of CHF, NT-proBNP >220 pg/ml, systolic dysfunction and diastolic impairment as measured by echocardiography (regardless of any CHF diagnosis) (different denominators are due to by differences in the number of subjects with missing data for HFREF and HFNEF).

Figure 4 shows the prevalence of the aforementioned four criteria used to define CHF among the study population (regardless of a CHF diagnosis). About two thirds (67%, 95%CI 60.8–72.9) of all women reported signs and symptoms suggestive of heart failure, such as breathlessness or fatigue, while less than half of all men (44%; 95%CI 39.9–49.0) reported such complaints. About 15% of all men or women showed a NT-proBNP serum concentration above 220 pg/ml. A reduced systolic function was found in 8% (95%CI 6.3–9.9) among men and in 5% (95%CI 3.1–6.4) among women. About one fourth of all women (23%, 95%CI 20.5–27.5) and almost one sixth of all men (15.5%, 95%CI 13.1–18.3) showed echocardiographic signs of an impaired diastolic function.

### Determinants

**Figure 5:**
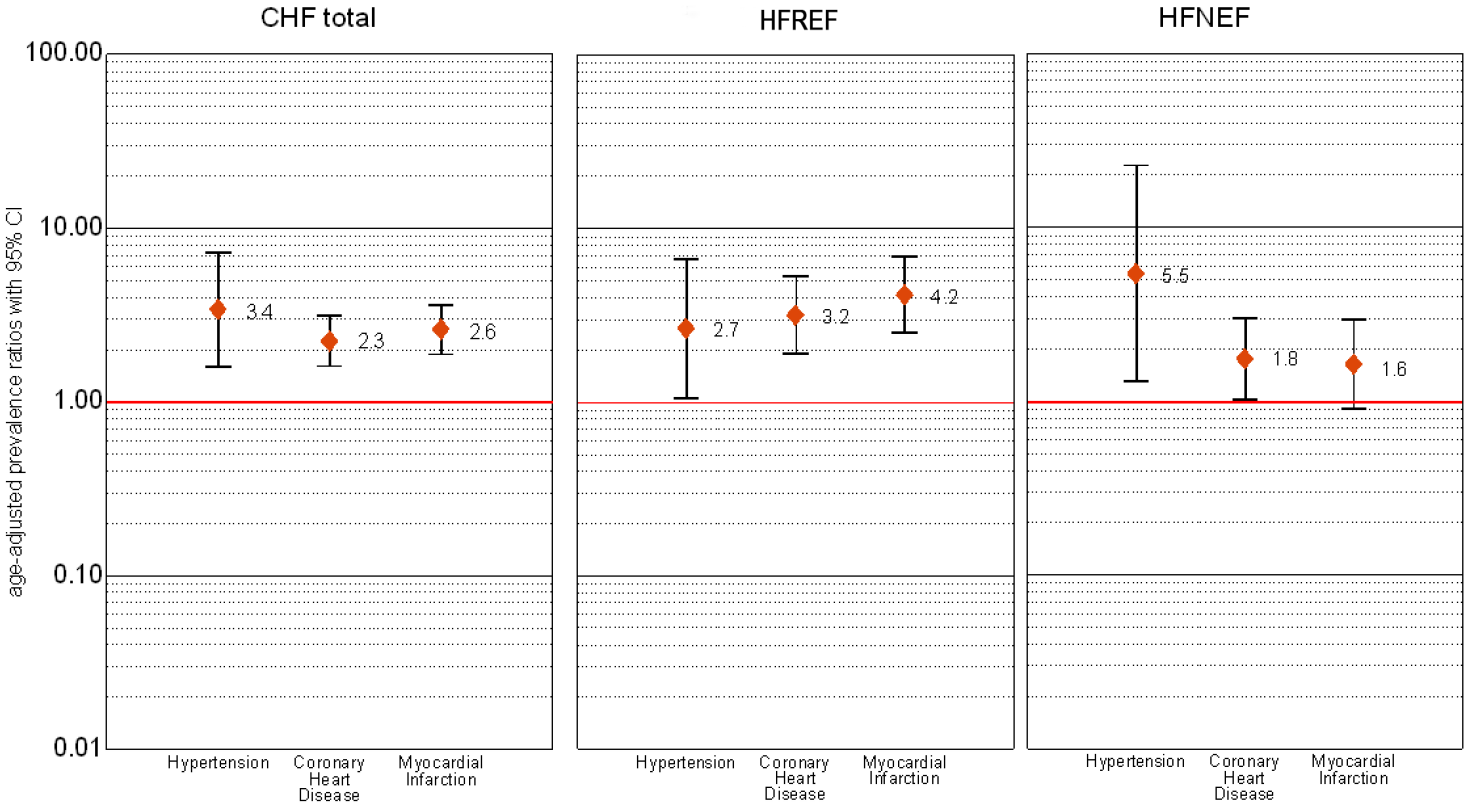
Prevalence ratio for the association of symptomatic HF (total, HFREF and HFNEF) with hypertension, CHD and myocardial infarction (adjusted for age and sex).

Among the prevalent cardiovascular diseases and risk factors that were analyzed as determinants of CHF, hypertension was the strongest determinant of CHF. The age- and sex-adjusted prevalence ratio (PR) for subjects with hypertension vs. subjects without hypertension was 3.4 (95%CI 1.6–7.3). For subjects with CHD versus subjects without CHD the age- and sex-adjusted PR was 2.3 (95%CI 1.6–3.1). Figure 5 shows the PRs for hypertension, CHD and myocardial infarction (MI) for all subjects with CHF as well as for those with SHF and HFNEF. While CHD or myocardial infarction as well as hypertension were strong determinants for HFREF, for HFNEF, the single strongest determinant was hypertension. While we could observe an association between diabetes and HFNEF for men with an age-adjusted PR of 2.0 (95%CI 1.1–4.0), for women we found an association only between diabetes and HFREF (PR 2.5, 95%CI 1.1–5.4). Body mass index (BMI) was associated with overall CHF for both sexes (PR 1.08 (95%CI 1.05–1.11)).

## Discussion

The CARLA Study was initiated to investigate the prevalence and incidence of cardiovascular diseases and their determinants in an elderly general population. One of the main goals of this investigation was to describe the burden of CHF, as it is one of the most important public health concerns.

As the data suggest, the study population can be described as a high-risk population with a high prevalence of cardiovascular risk factors such as hypertension, obesity, low physical activity, or high proportion of smokers. This is in accordance with other investigations comparing the prevalence of cardiovascular risk factors among different regions in Germany [29], [30]. The reasons for this high-risk profile compared with other German regions still remain unclear. One possible explanation might be the relatively poor socio-economic situation of the city of Halle, resulting in a high unemployment rate (almost 18% in 2006), which is considered a determinant of cardiovascular risk factors [31], [32]. In turn, the high proportion of cardiovascular risk factors could explain the high prevalence of heart failure in this elderly population.

The results of the CARLA Study showed a high prevalence of symptomatic heart failure compared to other studies, for the examined population aged 45 years and over [9]. For example, in contrast to the data of the Rotterdam Study [11] or the Olmsted County Study [12], the age-specific prevalence is noticeably higher in this East-German population (see Table 3). On the other hand, comparing the prevalence of systolic dysfunction (in this case defined as an EF less than 40%) between the CARLA Study and the Olmsted County Study the prevalence was quite comparable except for the highest age group. This adds further evidence that subjects with a high NYHA class of heart failure (especially women) are underrepresented in our study. However, among these studies different diagnostic approaches were used. While in the Olmsted County Study medical records were reviewed for the definition of heart failure in order to identify CHF diagnosis according to the Framingham Criteria [12], in the Rotterdam Study subjects were clinically examined in order to identify symptoms suggestive of CHF (e.g. shortness of breath, ankle oedema and pulmonary crepitations) [11]. As in the CARLA Study, both studies performed echocardiography. One possible explanation for the high prevalence of CHF (especially of HFNEF) is the use of NT-proBNP as diagnostic criterion in our study. Due to the diagnostic value of BNP as well as NT-proBNP we believe that we were able to identify a certain proportion of unrecognized HFNEF especially in subjects with symptoms suggestive of pulmonary diseases. This may lead to a truely higher prevalence compared with other population-based studies because they did not use NT-proBNP to define heart failure. Since the current ESC guideline on heart failure [33] recommend a NT-proBNP cutoff of 125 pg/ml, our diagnostic approach might be considered conservative and, therefore, an overestimation of the heart failure prevalence is unlikely. However, further studies are needed to compare our findings with other populations.“

**Table 3:** Prevalence of Heart Failure in the CARLA Study compared with other population-based studies.

		CARLA Study	Rotterdam Study[11]	Olmsted County Study[12]
		(Germany)	(The Netherlands)	(USA)
45–54 years		3.0		0.7%
55–64 years		6.0	0.7%	1.3%
65–74 years		10.4	2.7%	1.5%
75–84 years		22.0	13%	8.4%

In the present study, women, especially in the higher age-groups, were more likely to suffer from heart failure than men. This fact is also in accordance with the data from above mentioned studies [11], [12]. Sex differences regarding CHF are mainly due to a higher proportion of HFNEF among women. This observation may be related to the different aetiology of CHF between both sexes. Women are more likely to have hypertension as the aetiology of CHF, whereas for men coronary heart disease (including myocardial infarction) constitutes an important risk factor for CHF. Therefore, the prognosis for women with CHF is considered to be better than for men, since survival is worse for subjects with ischemic cardiomyopathy [34].

We found a strong correlation of CHF with age with a noticeable increase in CHF prevalence after the age of 70. The age-specific increase of CHF was similar in subjects with HFNEF compared to those with SHF. However, we observed a stronger age-specific increase of HFNEF as well as SHF in women compared with men.

Self-reported symptoms suggestive of CHF (dyspnoea, fatigue and oedema) were very common in the examined population, with an age-standardized prevalence of 44% in men and almost 67% in women. These symptoms were only self-reported during the standardized interview, and not verified by a clinical examination. This raises the question, whether the high prevalence of self-reported symptoms might have contributed to the high prevalence of chronic heart failure in the studied population. According our data, the relation between symptomatic heart failure and asymptomatic (preclinical) heart failure is very comparable to that in previous studies [12], [35], [36]. Therefore, we do not to believe, that the high prevalence of heart failure symptoms has artificially increased the CHF prevalence. However, several of these symptoms, especially dyspnoea, can also be found in pulmonary disease (mainly chronic obstructive pulmonary disease (COPD), obesity, and depression [37]. Therefore, a meaningful proportion of such symptoms cannot be attributed to cardiac failure. In our sample, 85% of all subjects with self-reported physician-diagnosed COPD reported symptoms which are typically also found in heart failure, and almost two thirds of all subjects with a BMI of 30 kg/m^2^ or more reported such complaints. While the age-standardized prevalence of COPD was quite similar between the sexes, we could observe a higher prevalence of obesity among women. This might be one possible explanation for the noticeably higher prevalence of symptoms suggestive of CHF among women.

The differentiation between COPD and CHF is complicated by the overlap of signs and symptoms. Furthermore, the accuracy of the echocardiography can be decreased due to hyperinflated lungs. However, the diagnosis HFNEF should be considered in subjects with COPD when abnormal LV mass or left atrial enlargement exist [38]. Evidence exist that there is a noticeable proportion of subjects with unrecognized CHF among subjects with COPD, which is estimated to be about 20% [39]. In our study population, the prevalence of HFREF and HFNEF was 10% and 8%, respectively, among all subjects with self-reported COPD. The prevalence of HFREF and HFNEF among all subjects taking antiobstructive medication (ATC R03) was 12% and 8%, respectively. These findings may lead to the assumption that a noticeable proportion of subjects with CHF and concomitant COPD exist.Nevertheless, we used elevated levels of NT-proBNP as another objective prerequisite for the diagnosis of heart failure. Therefore, cardiac dysfunction is the most likely explanation for heart failure symptoms even in subjects with, e.g., COPD or obesity as comorbidities. Interestingly, the mean NT-proBNP level in the subjects with HFNEF in the CARLA Study (779.7 pg/ml (SD 949.7)) was far higher than the baseline level in the I-PRESERVE trial, that tested the treatment of the AT-1 receptor antagonist irbesartan in HFNEF [40] with 354 pg/ml and where the majority of patients were in NYHA III functional class. This provides further supporting evidence that the algorithm we used in the CARLA-study actually identified subjects with heart failure as the main reason for their symptoms.

### Determinants

The aetiology of heart failure, especially of SHF, is fairly well understood. However, there is a lack of information on the aetiology of HFNEF and there is a need to understand the underlying pathophysiology of HFNEF, in order to provide effective treatment and improve outcomes for subjects with HFNEF. Up to now, there is no pharmacologic therapy which has shown to be effective in improving outcomes in patients with HFNEF. Trials like the I-PRESERVE-trial did not show any benefit to the patients [41]. Other studies showed only a weak benefit of pharmacologic therapy for patients with HFNEF in improving outcomes [42]. However, hypertension can be considered as the leading cause for the development of HFNEF [43]–[45]. Our analysis supports this hypothesis, showing a strong association between HFNEF and hypertension among both sexes. The results of our investigation underline that an appropriate treatment of hypertension may be crucial for prevention of HFNEF. However, there is a lack of evidence that a decline in HFNEF prevalence will go along with a decline in hypertension prevalence. Hence, prospective data are needed to describe the progression from preclinical diastolic impairment to the onset of symptomatic CHF in subjects with hypertension. Therefore, it is important to assess what proportion of subjects in our study population with preclinical diastolic dysfunction have progressed to HFNEF during the 4-year follow-up of the CARLA-Study that has recently been completed [19]. Besides hypertension as a main determinant, myocardial infarction and coronary heart disease were strongly associated with SHF but not with HFNEF. These findings are consistent with the already known pathophysiology of heart failure [41], [46]. Furthermore, our data suggest that the determinants of CHF in this population seem to be related to factors constituting metabolic syndrome such as diabetes or obesity.

### Strengths and limitations

The results of this study may be considered representative for the general population aged 45 to 85 years since a random sample from the population registry of the city of Halle had been selected, and a high participation rate could be achieved. However, lower participation rates among subjects too ill to attend the 4-hour examination resulted in a moderate selection bias towards a healthier population. Unfortunately, we do not have any information on chronic heart failure from subjects who refused to participate in the study. However, non-respondents reported a higher prevalence of cardiovascular diseases and adverse risk factors than study participants [20] which may have led to an underestimate of the true prevalence and risk of heart failure in the underlying population.

The main strengths of our study are the representative sample and the highly standardized assessment of the data (e.g. blood pressure measurement, ECG-recording, echocardiography) in agreement with other German and international studies. For the definition of heart failure, we were able to include information on symptoms, biomarker NT-proBNP, and echocardiographic parameters. The use of natriuretic peptides is recommended in addition to clinical information to detect diastolic dysfunction especially in high-risk populations [47]. One limitation was, however, that the symptoms suggestive of heart failure as well as different disease prevalences (e.g. CHD, COPD, diabetes) were self-reported and therefore we are not able to validate this information. However, the diagnosis of prevalent myocardial infarction was not only based on self-reported history of MI, but also on Minnesota-coded ECG that was validated by cardiologists. Regarding the study population, we cannot rule out the possibility of a selection bias. Furthermore, due to the cross-sectional nature of the data, caution must be exercised in the interpretation of the results, especially concerning the association with potential determinants.

## Conclusion

In summary, we found a higher prevalence of overall HF in this elderly population compared with previous population-based studies, which is primarily due to the higher prevalence of HFNEF, since the prevalence of systolic dysfunction is within the range of these population-based studies except for the highest age-groups. Our results underline the need for further elucidation of determinants of diastolic dysfunction and its progression to HFNEF in order to develop the prevention strategies that are needed to address the heart failure epidemic in the ageing population. Therefore, it will be important to assess what proportion of subjects with preclinical diastolic dysfunction have developed diastolic heart failure during the 4-year follow-up of the CARLA Study that has recently been completed [19].

To our knowledge, this is the first investigation in Germany describing the prevalence of symptomatic CHF among the general population and, in addition, the first study in Europe to describe the prevalence of symptomatic HFNEF.
